# Safety and efficacy of Ayurvedic interventions and Yoga on long term effects of COVID-19: A structured summary of a study protocol for a randomized controlled trial

**DOI:** 10.1186/s13063-021-05326-1

**Published:** 2021-06-03

**Authors:** Babita Yadav, Amit Rai, Pallavi Suresh Mundada, Richa Singhal, B. C. S. Rao, Rakesh Rana, Narayanam Srikanth

**Affiliations:** Central Council for Research in Ayurvedic Sciences Headquarters, 61-65, Institutional Area, Opp. ‘D’ Block, Janakpuri, New Delhi, 110058 India

**Keywords:** Ashwagandha, Agastya Haritaki, Post-COVID, long-COVID, Respiratory function, SARS-CoV-2, Yoga, WHO, randomised controlled trial, protocol, COVID-19

## Abstract

**Objectives:**

Primary Objective

• To assess the efficacy of Ayurveda interventions and Yoga in rehabilitation of COVID-19 cases suffering with long term effects of COVID 19 as compared to WHO Rehabilitation Self-Management after COVID-19- Related Illness.

Secondary Objective

• To assess the safety of Ayurvedic interventions in cases suffering with long term effects of COVID 19

**Trial design:**

Multi-centric, randomized, controlled, parallel group, open-label, exploratory study. The study duration is 9 months and the intervention period is 90 days from the day of enrolment of the participant.

**Participants:**

Patients of either sex between 18 to 60 years, ambulatory, willing to participate, with history (not more than 4 weeks) of positive RT-PCR for COVID-19 or IgM antibodies positivity for SARS CoV-2, but having negative RT-PCR for COVID-19 at the time of screening will be considered eligible for enrolment in the study. Critically ill patients with ARDS (acute respiratory distress syndrome), requiring invasive respiratory support in the intensive care unit, known case of any malignancy, immune-compromised state (e.g. HIV), diabetes mellitus, active pulmonary tuberculosis, past history of any chronic respiratory disease, motor neuron disease, multiple sclerosis, stroke, impaired cognition, atrial fibrillation, acute coronary syndrome, myocardial infarction, severe arrhythmia, concurrent serious hepatic disease or renal disease, pregnant or lactating women, patients on immunosuppressive medications, history of hypersensitivity to the trial drugs or their ingredients, depressive illness (before COVID-19), diagnosed psychotic illnesses, substance dependence or alcoholism will be excluded.

The trial will be conducted at two medical colleges in Maharashtra, India.

**Intervention and comparator:**

**Intervention Arm (Group-I):** Ayurveda interventions including Agastya Haritaki six gram and Ashwagandha tablet 500 mg twice daily orally after meals with warm water and two sessions of yoga (morning 30 minutes and evening 15 minutes) daily for 90 days, as per the post-COVID-19 care protocol provided in National Clinical Management Protocol based on Ayurveda and Yoga for management of COVID-19 published by Ministry of AYUSH, Government of India.

**Comparator Arm (Group-II):** WHO Rehabilitation Self-Management after COVID-19 related illness for 90 days.

The trial drugs are being procured from a GMP certified pharmaceutical company.

**Main outcomes:**

Primary Outcome: Change in respiratory function to be assessed by San Diego shortness of breath Questionnaire, 6-minutes walk test and pulmonary function test.

Secondary Outcomes:

- Change in High-resolution Computed Tomography (HRCT) Chest
- Change in Fatigue score assessed by Modified Fatigue Impact Scale
- Change in Anxiety score assessed by Hospital Anxiety and Depression Scale Score
- Change in Sleep Quality assessed by Pittsburgh Sleep Quality Index
- Change in the quality of life assessed by COV19-QoL scale
- Safety of the interventions will be assessed by comparing hematological and biochemical investigations before and after the intervention period and Adverse Event/ Adverse drug reaction

**Timelines for Outcome assessment:**

Subjective parameters and clinical assessment will be assessed at baseline, 15^th^ day, 30^th^ day, 60^th^ day and 90^th^ day. Laboratory parameters (CBC, LFT, KFT, HbA1c, Hs-CRP, D-dimer), Pulmonary function test and HRCT Chest will be done at baseline and after completion of study period i.e. 90^th^ day.

**Randomisation:**

Statistical package for Social Sciences (SPSS) version 15.0 is used to generate the random number sequences. The participants will be randomized to two study groups in the ratio of 1:1.

**Blinding (masking):**

The study is open-label design. However, the outcome assessor will be kept blinded regarding the study group allocation of the participants.

**Numbers to be randomised (sample size) Sample size:**

The sample size for the study is calculated assuming improvement in 6-minutes walk test by 40 meter in Group I and a change of 10 meter in Group II with a standard deviation of 50 meter based on the results of the previous studies, with 95% Confidence Level (α = 0.05) and 80% power and expecting a dropout rate of 20%. The number of participants to be enrolled in the study should be approximately 55 in each group. Hence, a total of 110 participants will be enrolled in the trial at each study site.

**Trial Status:**

Participants’ recruitment started on 1^st^ May 2021. Anticipated end of recruitment is August 2021. Protocol number: CCRAS-01 Protocol version number: 1.1, 13th January 2021.

**Trial registration:**

The trial is prospectively registered with the Clinical Trial Registry of India (CTRI) on 03^rd^ March 2021 [CTRI/2021/03/031686].

**Full protocol:**

The full protocol is attached as an additional file, accessible from the Journal website (Additional file 1). This communication serves as a summary of the key elements of the full protocol.

**Supplementary Information:**

The online version contains supplementary material available at 10.1186/s13063-021-05326-1.

## Supplementary Information

**Additional file 1:** Full protocol of Ayurveda interventions and Yoga in Long Covid.

## Data Availability

Raw data will be generated at study centres will be processed to the derived data supporting the findings of this study at CCRAS headquarters. It will be available from the corresponding author [BY] upon reasonable request.

