# Supplementary material for: Safety and efficacy of Ayurvedic interventions and Yoga on long term effects of COVID-19: A structured summary of a study protocol for a randomized controlled trial
Source: Trials. 2021 Jun 3;22:378. doi: 10.1186/s13063-021-05326-1 (PMC8173507; doi:10.1186/s13063-021-05326-1)
Supplement: Supplementary file 1 — Additional file 1: Full protocol of Ayurveda interventions and Yoga in Long Covid. [file 13063_2021_5326_MOESM1_ESM.docx]

**POST-COVID STUDY PROTOCOL**

**Title of the Study:** A Randomized controlled trial to evaluate the efficacy of Ayurvedic interventions (Agastya Haritaki and Ashwagandha) and Yoga in long term effects of COVID-19

**Trial Registration:** The trial is prospectively registered with the Clinical Trial Registry of India [CTRI/2021/03/031686].

**Protocol version**: CCRAS-IMR-Post COVID:1.1 dated 13^th^ January 2021

**Funding**: Central Council for Research in Ayurvedic Sciences, Ministry of AYUSH, Government of India, New Delhi.

**Roles and Responsibilities:**

**Protocol Contributors:** BY conceived the study. PM and AKR initiated the study design. Protocol was finalized with inputs from BCSR and NS. RR provided the statistical inputs. All authors contributed to refinement of the study protocol.

**Contact details of Trial Sponsor:**

**Role of Study Sponsor:** The funding agency has designed this study and will analyse the data and publish the results. However, it is not involved in the manufacturing and commercialization of the trial drugs as both the drugs are classical Ayurveda formulations.

**Co-ordination centre:** Central Council for Research in Ayurvedic Sciences, Jawahar Lal Nehru Bhartiya Chikitsa Evam Homoeopathy Anusandhan Bhawan, 61-65, Institutional Area, Opposite 'D' Block, Janakpuri, New Delhi-110058, INDIA

**Data Management:** CCRAS’s Central Biostatistical Monitoring Unit and the technical officers those are directly involved in this project

**This protocol is written by Central Council for Research in Ayurvedic Sciences, Ministry of AYUSH, Government of India, New Delhi. No part of this shall be published without written permission of the Head of the Council (**[**dg-ccras@nic.in**](mailto:dg-ccras@nic.in)**)**

**Background and Rationale**

Most people with COVID-19 experience mild symptoms or moderate illness. Approximately 10-15% of cases progress to severe disease, and about 5% become critically ill. Typically people recover from COVID-19 after 2 to 6 weeks. For some people, some symptoms may linger or recur for weeks or months following initial recovery. This can also happen in people with mild disease. People are not infectious to others during this time but are unable to return to their normal life. Some patients develop medical complications that may have lasting health effects. This prolonged illness after COVID-19, are seen even in young adults without underlying co-morbidities. There are many case reports from people who do not regain their previous health for a longer period following COVID-19. Risk factors for persistence of symptoms could be hypertension, obesity and mental health conditions.^1^

Little is known about the clinical course of COVID-19 following milder illness. Lingering symptoms reported by participants of a multi-state phone study in the USA included fatigue, cough, congestion or shortness of breath, loss of taste or smell, headache, body ache, diarrhea, nausea, chest or abdominal pain and confusion. In this survey of symptomatic adults who had a positive outpatient test result for SARS-CoV-2, 35% had not returned to their usual state of health when interviewed 2–3 weeks after testing. 20% (1 in 5) among those 18 to 34 years in good health, reported that some symptoms were prolonged and symptoms least likely to have resolved included cough, fatigue and shortness of breath. This report indicates that even among symptomatic adults tested in outpatient settings, it might take weeks for resolution of symptoms and return to usual health.^2^ In contrast, over 90% of outpatients with influenza recover within approximately 2 weeks of having a positive test result.^3^ Factors like old age and presence of multiple co-morbidities among adults hospitalized with COVID-19 are also associated with prolonged illness in an outpatient population. Notably, convalescence can be prolonged even in young adults without chronic medical conditions, potentially leading to prolonged absence from work, studies, or other activities.^2^

The previous corona virus outbreaks of SARS in 2002 and MERS in 2012 has reported reduced lung function and exercise capacity at six months post-discharge and post-traumatic stress disorder, depression, anxiety and reduced quality of life at one year were observed. A prospective cohort study performed on the long term effects of severe acute respiratory syndrome (SARS) epidemic emerged in 2003 showed that, 52% of SARS survivors had persistent impairment in diffusing capacity of the lungs for carbon monoxide and that exercise capacity and health status were significantly lower than the normal controls of the same age groups at 24 months post-illness.^3^ Another study, revealed that 40% of people recovering from SARS still had Psychiatric morbidities and chronic fatigue symptoms even 3.5 years after being diagnosed and so optimization of the treatment of mental health morbidities by a multidisciplinary approach with a view for long-term rehabilitation especially targeting psychiatric and fatigue problems, would be needed in the survivors.^4^

COVID-19 is presenting with a wide spectrum of clinical symptoms like severe respiratory dysfunction, septic shock and multiple organ dysfunction or failure etc. Body systems and organs that can be affected after the COVID-19 infection are heart (damage to heart muscle, heart failure), lungs (damage to lung tissue and restrictive lung failure), brain and the nervous system [loss of sense of smell (anosmia)], consequences of thrombo-embolic events such as pulmonary embolism, ischemic heart disease, stroke, cognitive impairment (e.g. memory and concentration), affected mental health (anxiety, depression, post-traumatic stress disorder and sleep disturbance), musculoskeletal symptoms (pain in joints and muscles) and fatigue are common conditions observed.^1^The problems faced by survivors of COVID-19 after recovery are getting reported now. More time and research is needed to understand the long-term effects of COVID-19, why symptoms persist or recur, how these health problems affect patients, the clinical course and likelihood of full recovery.A study has reported that high proportion of individuals of COVID-19 recovered reported fatigue, dyspnoea, joint pain and chest pain.^5^Health-related quality of life was poor among COVID-19 patients at the 1 month follow-up and patients suffered from significant physical and psychological impairment.^6^ Therefore, prospective monitoring of individuals exposed to SARS-CoV-2 is needed in order to fully understand the long-term impact of COVID-19, as well as to inform prompt and efficient interventions to alleviate suffering. Also there is an urgent need to address the issues of survivors of COVID-19 for their rehabilitation in terms of disease/ treatment induced complications, secondary prevention, mental/ psychosocial impact, and overall quality of life. As per the National Clinical Management Protocol based on Ayurveda and Yoga for management of COVID-19 issued by Ministry of AYUSH, Govt. of India, Ayurvedic *Rasayana* such as Ashwagandha and Yoga have been suggested for post-COVID management. Noticeably, the combination of Agastya Haritaki and Ashwagandha along with Yoga have the advantages of simplicity, affordability, and may be easily acceptable among society to fight against post COVID-19 condition. So this study has been designed to see the efficacy of Ayurveda interventions and Yoga in rehabilitating the patients suffering from prolonged effects of COVID-19.

**Objective(s)**

Primary Objective

- To assess the efficacy of Ayurveda interventions and Yoga in rehabilitation of COVID-19 cases suffering with long term effects of COVID-19 as compared to WHO Rehabilitation Self-Management after COVID-19- Related Illness

Secondary Objective

- To assess the safety of the Ayurvedic interventions in cases suffering with long term effects of COVID-19

**Study Design**

- Open label Randomized Controlled Multi-centric Trial with a study period of 90 days from the day of enrolment.
- 2-arm, parallel group exploratory study with 1:1 allocation ratio

**Study Setting**

- Two Academic Hospitals situated in Maharashtra, India viz. Government Medical College and Hospital, Nagpur and Datta Meghe Institute of Medical Sciences (Deemed as University),Wardha

**Eligibility Criteria**

**Inclusion criteria:** Patients of either sex between 18 to 60 years, ambulatory, willing to participate, with history (not more than 4 weeks) of positive RT-PCR for COVID-19 or IgM antibodies positivity for SARS CoV-2, but having negative RT-PCR for COVID-19 at the time of screening will be considered eligible for enrolment in the study.

**Exclusion criteria:** Critically ill patients with ARDS (acute respiratory distress syndrome), requiring invasive respiratory support in the intensive care unit, known case of any malignancy, immune-compromised state (e.g. HIV), diabetes mellitus, active pulmonary tuberculosis, past history of any chronic respiratory disease, motor neuron disease, multiple sclerosis, stroke, impaired cognition, atrial fibrillation, acute coronary syndrome, myocardial infarction, severe arrhythmia, concurrent serious hepatic disease or renal disease, pregnant or lactating women, patients on immunosuppressive medications, history of hypersensitivity to the trial drugs or their ingredients, depressive illness (before COVID-19), diagnosed psychotic illnesses, substance dependence or alcoholism will be excluded.

**Interventions**

**Intervention Arm (Group-I):** Ayurveda interventions including Agastya Haritaki six grams and Ashwagandha tablet 500 mg twice daily orally after meals with warm water and two sessions of yoga (morning 30 minutes and evening 15 minutes) daily for 90 days, as per the post-COVID-19 care protocol provided in National Clinical Management Protocol based on Ayurveda and Yoga for management of COVID-19 published by Ministry of AYUSH, Government of India.

**Comparator Arm (Group-II):** WHO Rehabilitation Self-Management after COVID-19 related illness for 90 days.

The trial drugs are being procured from a GMP certified pharmaceutical company.

**Withdrawal Criteria:** Participant not willing to continue or non-compliant (less than 80% compliance) with the study procedure; participant develops life threatening complication or any other severe illness because of other pathology which requires urgent treatment; Adverse effect (AE)/ Adverse drug reaction (ADR) necessitating hospitalization. The decision to withdraw a participant from the trial will be informed to the Sponsor and the Ethics Committee within two working days with detailed justification.

**Compliance:** The drug compliance will be assessed at each visit during the follow-up by counting the number of empty containers returned and assessing the approximate quantity of medicines consumed by the patient. Also, drug compliance report form will be provided to the study participants which has to be filled by the participant and submitted to the investigators during the follow-up visit. Similarly, compliance regarding Yoga protocol and WHO Rehabilitation Self-Management guidelines will also be accessed through compliance report form provided to the study participants.

**Concomitant and Rescue Medication:** Participants registered under the trial will be instructed to avoid the use of any other drugs on their own for any ailment and to consult the investigators for any symptom or complaint, or if they feel anything unusual. The investigator will record any medication(s) he/she may prescribe to alleviate their ailments. To alleviate any emergency medical condition, the use of any rescue medication will be permitted as per the discretion of the Investigators. However, the same will have to be documented in the Case Record Form.

**Outcomes:**

Primary Outcome: Change in respiratory function to be assessed by San Diego shortness of breath Questionnaire, 6-minutes walk test and Pulmonary Function test.

Secondary Outcomes:

- Change in High-resolution Computed Tomography (HRCT) Chest
- Change in Fatigue score assessed by Modified Fatigue Impact Scale
- Change in Anxiety score assessed by Hospital Anxiety and Depression Scale Score
- Change in Sleep Quality assessed by Pittsburgh Sleep Quality Index
- Change in the quality of life assessed by COV19-QoL scale
- Safety of the interventions will be assessed by comparing hematological and biochemical investigations before and after the intervention period and Adverse Event/ Adverse drug reaction

**Timelines for Outcome assessment:** Subjective parameters and clinical assessment will be assessed at baseline, 15^th^ day, 30^th^ day, 60^th^ day and 90^th^ day. Laboratory parameters (CBC, LFT, KFT, HbA1c, Hs-CRP, D-dimer), Pulmonary function test and HRCT Chest will be done at baseline and after completion of study period i.e. 90^th^ day.

**Participant Timeline:**

|  | **Study Period** | | | | | |
| --- | --- | --- | --- | --- | --- | --- |
|  | **Enrolment** | **Allocation** | **Post Allocation** | | | |
| **Time points** | **Screening** | **Baseline** | **15^th^ Day** | **30^th^ Day** | **60^th^ Day** | **90^th^ Day**  **(Close out)** |
| **Enrolment:** | | | | | | |
| Information and Informed consent |  |  |  |  |  |  |
| Eligibility evaluation |  |  |  |  |  |  |
| Allocation |  |  |  |  |  |  |
| Intervention: | | | | | | |
| Issue of Trial drug and Yoga Training to Group A |  |  |  |  |  |  |
| Training to practice ‘WHO Rehabilitation Self-Management after COVID-19’ to Group B |  |  | If required | If required | If required |  |
| Medical history and Demographic profile |  |  |  |  |  |  |
| Clinical examination |  |  |  |  |  |  |
| Prakriti (Constitution of body and mind) assessment |  |  |  |  |  |  |
| Assessment of Respiratory function, Fatigue, Sleep, Anxiety and Depression |  |  |  |  |  |  |
| Assessment of Quality of Life (COV19-QoL scale) |  |  |  |  |  |  |
| Laboratory and Radiological Investigations |  |  |  |  |  |  |
| Drug compliance |  |  |  |  |  |  |
| Rescue medication |  |  |  |  |  |  |
| Adverse events |  |  |  |  |  |  |

**Sample size:** The sample size for the study is calculated assuming improvement in 6-minutes walk test by 40 meter in Group I and a change of 10 meter in Group II with a standard deviation of 50 meter based on the results of the previous studies, with 95% Confidence Level (α = 0.05) and 80% power and expecting a dropout rate of 20%. The number of participants to be enrolled in the study should be approximately 55 in each group. Hence, a total of 110 participants will be enrolled in the trial at each study site.

**Recruitment:** The eligible participants will be screened from the patients with post COVID complaints visiting the Out-Patient Department of Government Medical College and Hospital, Nagpur and Datta Meghe Institute of Medical Sciences (Deemed as University),Wardha, Maharashtra, India.

**Randomization:** Statistical package for Social Sciences (SPSS) version 15.0 is used to generate the random number sequences. The participants will be randomized to two study groups in the ratio of 1:1.

**Allocation concealment:** The allocation concealment will be implemented through opaque, sealed envelopes. The allocation sequence will be generated by a statistician, who is not involved with the enrolment process.

**Blinding:** The study is open-label design. However, the outcome assessor will be kept blinded regarding the study group allocation of the participants.

**Data collection methods:** After informing the participants about the research study as per Patient Information Sheet, written consent (in triplicate) will be taken from the participants before screening them. After screening the participants, the eligible participants will be included in the study and data will be collected in a case record form. The data will be subsequently recorded in an e-format for statistical analysis and record.

**Data management:**

**Statistical methods:**

The categorical variables in the study data will be summarized as number (percentage) and compared using chi-square test. The continuous data having normal distribution will be represented as mean (SD), and data not following normal distribution as median (min-max). Parametric data will be analyzed by paired t-test and independent sample t-test for within and between group analysis respectively whereas non-parametric data will be calculated by Wilcoxon signed rank test and Mann-Whitney test for within and between group analysis respectively. All the data analysis will be done using the STATA software version 16.1.

**Data Monitoring:** The study will be monitored by Data and Safety Monitoring Board (DSMB). An Interim analysis, if required can be done when at least 25% participants have completed their trial period.

**Ethics Approval:** The study protocol has been approved by the institutional Ethics committees of both the study sites viz., Government Medical College, Nagpur, India and Datta Meghe Institute of Medical Sciences, Wardha, India on 3^rd^ February 2021 and 24^th^ February 2021 (vide EC-CT-2019-0129 and EC/Pharmac/GMC/NGP/2309) respectively. The study will be conducted in accordance with the principles of Declaration of Helsinki and the ICMR’s National Ethical Guidelines for Biomedical and Health Research on Human Participants (2017).

**Protocol Amendments:**

The trial will be conducted in compliance with the protocol. Deviations from the protocol will not be made except when necessary to alleviate an immediate hazard to trial patients. All the protocol amendments, including changes to interventions, examination, data collection and method of analysis will be reported to the sponsors and IEC at the earliest along with the exact reason.

**Consent:** Written informed consent will be taken from all the eligible and willing participants before their screening by the study investigators.

**Confidentiality:** All study-related information will be stored securely at the study site. All participant information will be stored in locked file cabinets in areas with limited access. All the data collected in case record forms will be entered in an e-format developed by the CCRAS headquarters. All records that contain names or other personal identifiers, such as locator forms and informed consent forms, will be stored separately from study records identified by code number. All local databases will be secured with password-protected access systems. Forms, lists, logbooks, appointment books, and any other listings that link participant ID numbers to other identifying information will be stored in a separate, locked file in an area with limited access. The data will be identified by a coded identification number only to maintain participant confidentiality. A Data Safety Monitoring Board will periodically monitor the process.

**Declaration of Interests:** There is no competing interest between the investigators. The funding agency has designed this study and will analyse the data and publish the results as both medicines are classical Ayurveda formulations. It has no role in manufacturing and marketing the trial drugs.

**Access to data:** Raw data will be generated at study centres will be processed to the derived data supporting the findings of this study at CCRAS headquarters.

**Ancillary and Post-trial care:** No ancillary studies are proposed with this trial. The participants will be given routine medical care if required, after completion of study period.

**Dissemination:** The Principle investigators at individual study centers will not report the data collected from their centers alone. All the presentations and publications will protect the integrity of the major objective(s) of the study. Data that break the blind will not be presented prior to the release of mainline results. Recommendations as to the timing of presentation of such endpoint data and the meetings at which they might be presented will be given by the CCRAS Headquarters.

**Appendices:** Informed Consent Form and Patient Information Sheet.
